# TAVI—thick on technology but thin on evidence

**DOI:** 10.1007/s12055-018-0731-2

**Published:** 2018-10-17

**Authors:** Kunal Sarkar

**Affiliations:** Medica Superspecialty Hospital, Kolkata, 700099 India

**Keywords:** Aortic valve, TAVI, Trial

## Abstract

Transcatheter aortic valve implantation (TAVI) has emerged as one of the most popularly deployed interventional innovations of recent times. After addressing the inoperable and high-risk patients, it is application is being extended to intermediate and low-risk category. There is some disquiet about the strength of evidence on which the clinical application is based. Durability and pacemaker requirement are also areas of concern. This review highlights the areas of concern on these aspects. There is also a need to address these deficiencies in future trials and also bring updated database reports in the public domain.

Since its inception in 2002 by Dr. Cribbier, transcatheter aortic valve implantation (TAVI) has become one of the most rapidly adopted medical innovations. As we continue to be impressed with the increasing number of procedures, the strength of evidence on which it is proliferating stands to scrutiny. Approval by Conformité Européenne (CE, Europe) was instrumental in its rapid adoption in Europe, particularly in Germany. CE assessment of a medical device is not very different from the assessment of consumer appliances, (e.g., toaster or a kettle). Hence, obtaining a CE approval is not a hallmark of quality for any medical device. European certification does not mandate clinical effectiveness and safety to be established on basis of randomized trials. CE’s list of requirement for medical devices relates to listing compliance. It is not an authentication of its evidence-based effectiveness [1, 2].

The fact that trials were at all designed were to address the requirement of obtaining approval for its use in the USA.

The explosive adoption in Europe was aided by the preferential reimbursement by the German insurance agencies for TAVI as compared to surgical aortic valve replacement (SAVR) and led to a rapid deployment of these valves [3].

The Sapien valve (Edwards Lifesciences) was the principal device on which the initial evaluation trial Partner-I was based [4]. This focused on the inoperable and surgical high-risk patients with a predicted Society of Thoracic Surgeons (STS) score more than 8–10% [4].

Patients who were deemed inoperable did better having received the Sapien valve than those who were only on medical treatment. The performance of the TAVI patients as compared to the high-risk SAVR group was also comparable, apart from a slightly increased incidence of strokes. Hence, Partner-I claimed breakthrough outcomes in those patients who were deemed either inoperable or high risk for SAVR. But, some salient deficiencies of the trial remained obscured from the public domain. Firstly, only 12% of all screened patients were randomized [5].

There were hardly any substantive differences between the inoperable and high-risk operable groups (Table 1). [6]

**Table 1:** PARTNER trial baseline characteristics [6]

	Cohort A	Cohort B
Medical characteristics		
NYHA class III or IV, %	94.2	93.1
Coronary artery disease, %	75.9	71.0
Previous myocardial infraction, %	28.4	22.5
Previous CABG, %	43.4	41.5
Previous PCI, %	33.3	27.7
Previous balloon aortic valvuloplasty	11.8	20.3
Cerebral vascular disease, %	28.4	27.5
Peripheral vascular disease, %	42.3	27.7
COPD, %: any	43.2	46.9
COPD, %: oxygen-dependent	8.2	23.5
Creatinine > 2 mg/dL, %	9.1	7.6
Atrial fibrillation, %	41.8	40.9
Previous pacemaker, %	21.0	21.2
Pulmonary hypertension, %	39.4	43.1
Liver disease, %	2.3	3.4
Moderate or severe mitral regurg., %	20.6	22.6
Frailty, %	16.6	23.1
Anatomical characteristics		
Extensively calcified aorta, %	0.9	15.1
Effects of chest-wall irradiation, %	0.9	8.7
Chest-wall deformity, %	0.2	6.7
Multiple previous interventions	NR	NR

*Cohort A* high-risk operable patients, *Cohort B* inoperable patients, *NR* not reported

Secondly, occurrence of comorbidities and frailty were not uniform in the TAVI and non-TAVI groups [7] in Cohort B. Incidence of comorbidities was noticeably higher in the surgical AVR group (Table 2) [6].

**Table 2:** Selection of patient characteristics in the PARTNER trial (inoperable patients) [6]

	TAVI (*n* = 179)	Standard therapy (*n* = 179)	*p* value
STS score	11.2 ± 5.8	12.1 ± 6.1	0.14
Logistic EuroSCORE	26.4 ± 17.2	30.4 ± 19.1	0.04
Coronary artery disease, *n* (%)	121 (67.6)	133 (74.3)	0.20
Previous myocardial infarction, *n*/total (%)	33/177 (18.6)	47/178 (26.4)	0.10
COPD (any)	74 (41.3)	94 (52.5)	0.04
Creatinine >2 mg/dL (177 μmol/l, *n*/total) (%)	10/178 (5.6)	17/178 (9.6)	0.23
Atrial fibrillation, *n*/total (%)	28/85 (32.9)	39/80 (48.8)	0.04
Frailty, *n*/total (%)	21/116 (18.1)	33/118 (28.0)	0.09
Mean LVEF, %	53.9 ± 13.1	51.1 ± 14.3	0.06
All anatomic inoperable patients*, *n* (%)	53 (29.6)	37 (20.7)	0.05
Extensively calcified aorta, *n* (%)	34 (19.0)	20 (11.2)	0.05
Deleterious effects of chest-wall irradiation, *n* (%)	16. (8.9)	15 (8.4)	1.00
Chest-wall deformity, *n* (%)	15 (8.4)	9 (5.0)	0.29

The estimated operative risk (EUROScore), chronic obstructive pulmonary disease (COPD), and atrial fibrillation were statistically significantly (*p* < 0.05) and more prevalent in the control group. There were also more patients with a previous myocardial infarction (26.4 vs. 18.6%, *p* = 0.10), and control patients had a lower left ventricular ejection fraction (LVEF) than TAVI patients (51.1% vs. 53.9%, *p* = 0.06). Patients with “frailty” were over represented in the standard therapy arm. There were also less patients with an extensively calcified aorta (11.2 vs. 190%, *p* = 0.05), i.e., those with a better outlook after a successful intervention, since they do not necessarily have severe medical comorbid conditions

*Data obtained from the study sponsor combining the patient characteristics “Extensively calcified aorta,” “Deleterious effects of chest-wall irradiation,” and “Chest-wall deformity”; Smith et al. [4]

Thirdly, the trial was clouded by ethical considerations [1]. The principal investigator appeared to have a conflict of interest, in having been a financial beneficiary from the industry [8].

Methodological and ethical issues prompted the United State Food and Drug Administration (USFDA) to ask for an extension of the trial to another 100 patients under more scrutinized environment—“The continued access study” (Table 3) [6].

**Table 3:** Unpublished results related to the PARTNER trial (inoperable patients) [6]

	Pivotal trial cohort b						Randomized continued access cohort b	
	Pivotal trial published		Anatomically inoperable subgroup		Medically inoperable subgroup		TAVI	Control
	TAVI	Control	TAVI	Control	TAVI	Control		
*n*	179	179	53	37	126	142	41	49
30-day mortality	5.0%	2.8%	1.9%	2.7%	6.3%	2.8%	9.8%	2.1%
1-year mortality	30.7%	50.7%	24.5%	52.4%	33.3%	50.3%	34.3%	21.6%

All data refer to intention-to-treat analyses. Pivotal trial data: Smith et al. [4]; Anatomically and medically inoperable sub-groups: personal communication, Edwads Lifesciences S.A., Switzerland (August 10, 2011); Continued Access Study: FDA (July 20, 2011) [8]

*n* number of patients per sub-group, *TAVI* transcatheter aortic valve implantation, *Control* “Standard Therapy”, including balloon aortic valvuloplasty in most patients, in combination with a medical supportive treatment

Continued Access data of Cohort B had significantly higher mortality and stroke rate, as compared to the results of Pivotal Partner Trial.

This extension of Partner-I seems to be a well-kept secret, and the data have been scarcely displayed or discussed [1]. There is only one recorded instance of the case study being discussed in a FDA meeting [9]. It is unsatisfactory that this study failed to find a mention as a data set that was markedly different from the data of the “Pivotal Partner Study” [10].

With some reluctance, USFDA revealed the data of 90 patients in the continued access study. TAVI group had a noticeably increased mortality and stroke rate. On queries by various clinical and research agencies, Edwards Life sciences have not been very forthcoming with the data. This provoked a sharp response from the British Medical Journal, “The Partner trial seems to have important problems; the most relevant being publication bias and lack of data transparency, unbalanced patient characteristics, and incompletely declared conflicts of interest” [1].

In a largely noncritical environment, Partner-I, as expected, gave way to the next trial of TAVI in the intermediate risk group, with STS score of 4–8% (Partner-II) [5].

The results of Partner-II showed comparable performance of TAVI both in terms of mortality and mortality. This led to the approval by USFDA for usage in USA. European guidelines also ratified the application to the intermediate risk group [11].

The lack of interrogation into the details of Partner-II was baffling. It defied the norms of prospective randomized trials as a large portion of the surgical cohort and was propensity matched from an earlier database [11, 12]. Even then, the propensity matching seemed distinctly biased towards a lower incidence of comorbidities and concomitant coronary bypass in the TAVI group [6, 13].

The chorus of disapproval into the methodology of Partner-II was met with indifference from the practice opinion makers. Approval of usage in the intermediate risk group remained unaffected [11]. The path was paved for Partner-III in the low-risk group and patient enrollment has also commenced [14].

Impetus from industry seems to have prevailed over the restraint that was necessary, based on clinical evidence.

Valve Research Academic Consortium (VARC) has published specific parameters on which procedural complications can be defined and detected. The essential definitions of complications and composite endpoints of device safety were summarized by VARC-2 (Table 4).

**Table 4:** Composite endpoints according to Valve Academic Research Consortium-2 (VARC-2) [15]

Device success	
► Absence of procedural mortality AND	
► Correct positioning of a single prosthetic heart valve into proper anatomical location AND	
► Intended performance of the prosthetic heart valve (no prosthetic-patient mismatch and mean aortic valve gradient < 20 mmHg or peal velocity < 3 m/s) AND	
► No moderate or severe prosthetic valve regurgitation	
Early safety (at 30 days)	
► All-cause mortality	
► All strokes (disabling and nondisabling)	
► Life-threatening bleeding	
► Acute kidney injury (stage 2 or 3, including renal replacement therapy)	
► Coronary artery obstruction requiring intervention	
► Major vascular complication	
► Valve-related dysfunction requiring repeat procedure (balloon valvuloplasty, transcatheter aortic valve implantation on (TAVI), or surgical aortic valve replacement)	
Clinical efficacy (after 30 days)	
► All-cause mortality	
► All strokes (disabling and nondisabling)	
► Hospitalizations for valve-related symptoms or worsening congestive heart failure	
► New York Hear Association (NYHA) class III or IV	
► Valve-related dysfunction (mean aortic valve gradient ≥ 20 mmHg, effective orifice area ≤ 0.9–1.1 cm^2^, and/or Doppler velocity index < 0.35 m/s) AND/OR	
► Moderate or severe prosthetic valve regurgitation	
Time-related valve safety	
► Structural valve deterioration	
► Valve-related dysfunction (mean aortic valve gradient ≥ 20 mmHg, effective orifice are ≤ 0.9–1.1 cm^2^, and/or Doppler velocity index < 0.35 m/s, and/or moderate or severe prosthetic valve regurgitation)	
► Prosthetic valve endocarditis	
► Prosthetic valve thrombosis	
► Thromboembolic events (e.g., stroke)	
► VARC bleeding unless clearly unrelated to valve therapy (e.g., trauma)	

In the recent past, data from the German Registry of Aortic Valve Replacement (GARY) has been made available. The database includes 3876 transcatheter aortic valve replacements and 9987 surgical aortic valve replacements with or without coronary bypass. The mortality ranged from 2.4 to 4.4% for SAVR (surgical AVR) and 5–8% for TAVI [15].

Vascular access-related complications occur in 16% of all transfemoral approach. This is in spite of significant reduction sheath sizes. Incidence of bleeding complications, which is considered an independent risk factor for mortality was between 0 and 14.9% [16]. Cardiac tamponade is also reported in 0.2–4.3% of cases. Balloon expandable prostheses are associated with 1.1% incidence of aortic root rupture.

Coronary obstruction though infrequent reported 0.2–0.4% of cases, mainly caused by the displacement of calcific leaflets [15].

Aortic regurgitation is an adverse prognostic indicator; this is either transvalvular or paravalvular. Aortic regurgitation occurred to some extent in excess of 60% of patients in Partner trial, 11.8% being moderate and 12.2% being severe [15]. In GARY registry, AR after transvascular access occurs in 62.8%, with 7% being moderate and 0.7% being severe [16].

Complications like postimplantation permanent pacemaker implantation and subclinical valve thrombosis are significantly higher in TAVI. Some reports suggest an incidence of subclinical valve thrombosis in excess of 12% [7, 17].

Occurrence of paravalvular leak is noticeably higher in TAVI as compared to SAVR. With design changes, the incidence is probably on the decline. This has introduced a new normal in aortic valve replacement—“acceptable para valve leaks” [7]. Data from the German database highlights 61% mortality, in patients with more than mild aortic regurgitation, within 1 year [18].

Incidence of postoperative paravalvular leak in surgical AVR is in the range of 1–5%. It can be corrected once it is detected with intraoperative transesophageal echocardiography.

Much of the data on severity of paravalvular leaks was subjective, due to lack of gradation norms. The subsequent grading of severity by Paravalvular Leak Academic Research Consortium (PVL-ARC) will help to have more objective assessment [19].

Post-TAVI conduction defects requiring permanent pacemaker implantation (PPI) is reported in most meta-analyses in 15–33.7% of cases [15, 20, 21]**.** In GARY registry, it was noted in 23.7% of transfemoral and 9.9% of transapical cases [15, 16].

Core valve device was associated with an incremental PPI requirement (25.2 vs. 3%).

Stroke rates have been lower than expected in most large series [9, 22]. The GARY database reports an in-hospital incidence of 1.8% with a 3% incidence at 30 days [15, 16].

The TAVI valve irrespective of the brand is a standard biological valve that was being implanted over past decades in SAVR. The atraumatic care that one adopts in course of surgical implantation is sacrificed for various types of tissue trauma in the course of per cutaneous implantation. This is expected to have a negative impact on its durability. This is substantiated by reports suggesting an accelerated structural attrition rate [23, 24].

This again questions the wisdom of permitting a trial in the lower age and lower risk group. Recent publication from Goldstone et al. makes a strong case for survival advantage for mechanical valves in the aortic position below 55 years of age [25].

The crying need for registry data has been somewhat addressed in recent years with GARY-(Germany), United States Trans Valvular Therapy (US TVT) (USA), FRANCE 2 (France), and United Kingdom (UK) TAVI Registry. As GARY had been mentioned in some detail, a brief synopsis of the other datasets is as follows (Table 5) [17–19, 26].

**Table 5:** Recent TAVI database, 30 day and 1-year mortality

Databases	30 day mortality	1-year mortality
German registry (GARY) [18]	5.2%	16.6%
US TVT registry [17]	7%	23.7%
UK TAVI registry [26]	6.3%	19.3%
FRANCE TAVI registry (France-II) [19]	9.5%	24.1%

These reports points towards an unexpectedly large attrition rate after the first year [26]. This trend is mirrored in the STS report of the US TAVI Registry and France 2 [17].

This a consequence of persisting procedural complications, probably the deleterious effect of persisting aortic incompetence.

Postprocedural survival in the octogenarians does not match the normal age-related survival after a successful TAVI. These points refer to persistence of problems that compromises the expected survival [13].

In times of extreme pressure on health care resources, irrespective of the economic stature of the country, adoption of a technology, which is 10 to 15 times more expensive than existing technology with inferior results begs serious scrutiny.

Judging by the Incremental Cost Effectiveness Ratio (ICCER) thresholds, TAVI seems a financial burden even in the USA, the UK, and countries of European Union (EU), and needless to say, an unbearable burden on the emerging Asian economies [12].

As we struggle to provide incremental resources in primary, secondary, and tertiary health care, embracing a vastly expensive option with inferior outcomes seems illogical.

Admiration for an innovation is well-acknowledged, but the evidence needs sincere scrutiny, based on the principles of evidence-based medicine. The chorus from the industry and interventionists has always been based on the exploding numbers and modifications in the devices. Respect for evidence and need for credible data seems to have taken a back seat for now. Are we bracing ourselves for distressed retrospective look back at inappropriate use of technology in not too distant future? A syndrome that is not totally alien to the device and pharmaceutical industry [16, 27–31].

This review runs the risk of being labeled as a Paleolithic protest against emerging technology. Still, it is being tabled with the conviction that respect for evidence and scientific methodology has not entirely been hypothecated to industry sponsored euphoria.

The collective conscience and intelligence of the cardiology and cardiac surgical community needs to navigate this disruption with clinical and economic probity.
